# Supplementary material for: Virulence and Antimicrobial Resistance Profiles of Salmonella enterica Serovars Isolated from Chicken at Wet Markets in Dhaka, Bangladesh
Source: Microorganisms. 2021 Apr 28;9(5):952. doi: 10.3390/microorganisms9050952 (PMC8145576; doi:10.3390/microorganisms9050952)
Supplement: Supplementary file 1 [file microorganisms-09-00952-s001.zip › microorganisms-1152112-supplementary/Supplementary File/Table S1.docx]

**Table S1. Primers used in the study to detect *Salmonella enterica* serovar and resistance gene**

| **PCR** | **Target Gene** | **Sequence** | **Amplicon size (bp)** | **References** |
| --- | --- | --- | --- | --- |
| m-PCR-1 | Typh | F-TTGTTCACTTTTTACCCCTGAA  R-CCCTGACAGCCG TTAGATATT | 401 | [51] |
|  | *sdf*1 and *sdf*-II | F-TGTGTTTTATCTGATGCAAGAGG  R-CGTTCTTCTGGTACTTACGATGAC | 293 | [50] |
| m-PCR-II | *bla*TEM | F- CATTTCCGTGTCGCCCTTATTC  R-CGTTCATCCATAGTTGCCTGAC | 800 | [58] |
|  | *bla*SHV | F-AGCCGCTTGAGCAAATTAAAC  R-ATCCCGCAGATAAATCACCAC | 713 | [58] |
|  | *bla*OXA | F-GGCACCAGATTCAACTTTCAAG  R-GACCCCAAGTTTCCTGTAAGTG | 564 | [58] |
| m-PCR-III | blaCTX-M-1 | F-TTAGGAARTGTGCCGCTGYA  R-CGATATCGTTGGTGGTRCCAT | 688 | [58] |
|  | blaCTX-M-2 | F-CGTTAACGGCACGATGAC  R-CGATATCGTTGGTGGTRCCAT | 404 | [58] |
|  | blaCTX-M-9 | F-TCAAGCCTGCCGATCTGGT  R-TGATTCTCGCCGCTGAAG | 561 | [58] |
|  | blaCTX-Mg8/25 | F-AACRCRCAGACGCTCTAC  R-TCGAGCCGGAASGTGTYAT | 326 | [58] |
| m-PCR-IV | *Sul1* | F-CGG CGT GGG CTA CCT GAA CG  R-GCC GAT CGC GTG AAG TTC CG | 433 | [59] |
|  | *Sul2* | F-CGG CGT GGG CTA CCT GAA CG  R-GCC GAT CGC GTG AAG TTC CG | 721 | [59] |
|  | *Sul3* | F-CAACGGAAGTGG GCGTTG TGGA  R-GCT GCA CCA ATT CGC TGAACG | 244 | [59] |
| m-PCR-V | *tet(A)* | F-GGC GGTCTT CTT CAT CATGC  R-CGG CAG GCA GAG CAA GTAGA | 502 | [59] |
|  | *tet*(B) | F-CGC CCA GTG CTG TTG TTGTC  R-CGC GTT GAG AAG CTG AGG TG | 173 | [59] |
|  | *tet*(C) | F-GCT GTAGGCATAGGCTTGGT  R-GCC GGA AGC GAG AAGAATCA | 888 | [59] |
|  | *strA/strB* | F-ATGGTGGACCCTAAAACTCT  R-CGTCTAGGATCGAGACAAAG | 893 | [59] |
