# Supplementary material for: Virulence and Antimicrobial Resistance Profiles of Salmonella enterica Serovars Isolated from Chicken at Wet Markets in Dhaka, Bangladesh
Source: Microorganisms. 2021 Apr 28;9(5):952. doi: 10.3390/microorganisms9050952 (PMC8145576; doi:10.3390/microorganisms9050952)
Supplement: Supplementary file 1 [file microorganisms-09-00952-s001.zip › microorganisms-1152112-supplementary/Supplementary File/Table S2.docx]

**Table S2.** **Primers used in the study to detect virulence gene of *Salmonella enterica* serovar**

| **PCR** | **Target Gene** | **Virulence factor** | **Sequence** | **Amplicon size (bp)** | **References** |
| --- | --- | --- | --- | --- | --- |
| u-PCR-1 | *inv*A | Invasion | F-GTGAAATTATCGCCACGTTCGGGCAA  R-TCATCGCACCGTCAAAGGAACC | 284 | [49] |
| u-PCR-2 | *agf*A | Fimbria | F-TCCACAATGGGGCGGCGGCG  R-CCTGACGCACCATTACGCTG | 350 | [60] |
| u-PCR-3 | *Ipf*A | Fimbria | F-CTTTCGCTGCTGAATCTGGT  R-CAGTGTTAACAGAAACCAGT | 250 | [61] |
| u-PCR-4 | *hil*A | Invasion | F-CTGCCGCAGTGTTAAGGATA  R-CTGTCGCCTTAATCGCATGT | 497 | [62] |
| u-PCR-5 | *siv*H | Invasion | F-GTATGCGAACAAGCGTAACAC  R-CAGAATGCGAATCCTTCGCAC | 763 | [63] |
| u-PCR-6 | *sef*A | Fimbria | F-GATACTGCTGAACGTAGAAGG  R-GCGTAAATCAGCATCTGCAGTAGC | 488 | [64] |
| u-PCR-7 | *sop*E | Effector protein | F-GGATGCCTTCTGATGTTGACTGG  R-ACACACTTTCACCGAGGAAGCG | 398 | [65] |
| u-PCR-8 | *spv*C | Plasmid | F-CCCAAACCCATACTTACTCTG  R-CGGAAATACCATCTACAAATA | 669 | [66] |
